# Clinical Assessment of Dry Eye Disease in Patients with Keratoconus in Saudi Arabia

**DOI:** 10.3390/healthcare13222890

**Published:** 2025-11-13

**Authors:** Saleh Alshammeri, Azzam Alharbi

**Affiliations:** Department of Optometry, College of Applied Medical Sciences, Qassim University, Buraydah 51452, Saudi Arabia

**Keywords:** keratoconus, dry eye, quality of life, health

## Abstract

**Purpose**: The purpose of this study was to investigate dry eye disease (DED) characteristics in patients with keratoconus (KC) using non-invasive methods. **Methods**: A total of 34 participants were included in the study. Corneal topography was conducted for each participant utilizing Pentacam, followed by grouping according to the results obtained. The patients with Kmax > 47.2 D (KC) were considered the keratoconus group (n = 17). Healthy control participants with Kmax < 47.2 D were considered the control group (n = 17). Non-invasive tear breakup time (NITBUT), tear meniscus height (TMH), (OSDI) questionnaire, meibography, and lipid layer evaluation were assessed across the groups. **Results**: Dry eye assessment revealed that the mean non-invasive tear breakup time (NITBUT) was significantly shorter in the keratoconus group (9.88 ± 3.25 s) compared to the control group (18.94 ± 4.26 s, *p* < 0.001). Tear meniscus height was also reduced in the keratoconus group (0.185 ± 0.053 mm) versus the control group (0.358 ± 0.076 mm, *p* < 0.001). OSDI scores were higher in the keratoconus group (21.41 ± 5.93) compared to the control group (7.52 ± 5.05, *p* < 0.001), reflecting greater subjective dry eye symptoms. Meibography showed more severe Meibomian gland dropout in the keratoconus group (2.11 ± 0.60 vs. 0.70 ± 0.34, *p* < 0.001). Lipid layer evaluation showed no significant difference between groups (*p* = 0.070). **Conclusions**: These findings suggested a significant correlation between keratoconus and dry eye disease, with keratoconus patients showing reduced tear film stability, decreased tear volume, and increased meibomian gland dysfunction. While lipid layer changes were not significant, the results emphasized the need for comprehensive evaluation of ocular surface parameters in keratoconus management.

## 1. Introduction

Keratoconus (KC) is chronic, non-inflammatory corneal ectasia, which is associated with decreased visual acuity secondary to corneal steepening, irregular astigmatism, and central corneal scarring [1]. Several studies have demonstrated an increased risk of dry eye disease in patients with keratoconus. For instance, a cross-sectional study was performed in 2017–2018 to investigate the ocular surface alterations in patients with mild or severe KC and patients without KC. The research discovered that the mean scores of the Ocular Surface Disease Index (OSDI) were significantly higher and the mean values of TBUT and the Schirmer-I test were lower in patients with [2]. In another study, a pilot, experimental, prospective study conducted in 2013 compared the signs and symptoms of dry eye in KC patients versus healthy subjects. They found that KC patients suffer greater symptoms of dry eye and greater tear instability [3]. Dry eye disease (DED) is a multifactorial disease of the ocular surface characterized by a loss of homeostasis of the tear film and accompanied by ocular symptoms, in which tear film instability, hyperosmolarity, ocular surface inflammation and damage, and neurosensory abnormalities play etiological roles [4]. Another study in Saudi Arabia found that the prevalence of KC varies geographically, with a prevalence rate of 8.59% reported in Taif [5], while in this study, they found that the incidence of (KC) in Asir Province is 20 cases per 100,000 populations [6], and the prevalence of pediatric (KC) is 5.56% [7]. Additionally, a study found that overall prevalence of DED in Saudi Arabia is estimated to be 49.5% [8]. Dry eye disease in patients with KC has undergone thorough investigation in recent years. Keratography helps to accurately assess dry eye disease (DED). Studies have focused on assessing the correlation between dry eye disease (DED) in patients with KC using invasive methods such as the Schirmer test and vital dye assessments to investigate the relationship between DED and KC [2,3]. Given the lack of regional data using non-invasive techniques, this study aims to evaluate DED in KC patients in Saudi Arabia through non-invasive diagnostic methods and we expect to provide valuable insights into the relationship between KC and DED in the Saudi population.

## 2. Methodology

This cross-sectional observational study was designed to investigate and compare the prevalence and severity of DED in Saudi residents with and without KC. A representative sample of Saudi residents aged 18 to 35 years was selected, comprising 34 participants divided into two equal groups. The KC group included 17 participants diagnosed with the condition, while the control group consisted of 17 participants without KC. Participants were matched by sex and age to reduce confounding effects. Grouping of participants was based on Kmax values obtained from Pentacam measurements for the eye with the higher Kmax [9]. Individuals with Kmax greater than 47.2 D were classified as the KC group, while those with Kmax less than 47.2 D were included in the control group [10].

Participants in the KC group were recruited from Qassim University (QU) Hospital, where their diagnosis was confirmed by corneal topography. In contrast, the control group was recruited through random sampling of individuals at (QU).

The inclusion criteria for this study required participants to be 18 years or older, with a confirmed diagnosis of KC (for the KC group) and the ability to provide informed consent. Participants were excluded if they had a history of ocular surgery (except corneal cross-linking performed within the past year period), corneal infections, systemic diseases that could affect the eyes, systemic autoimmune diseases (e.g., Sjögren’s syndrome), pre-existing or ongoing treatment for DED, or if they were using topical ocular anti-inflammatory medications.

**Figure 1:**
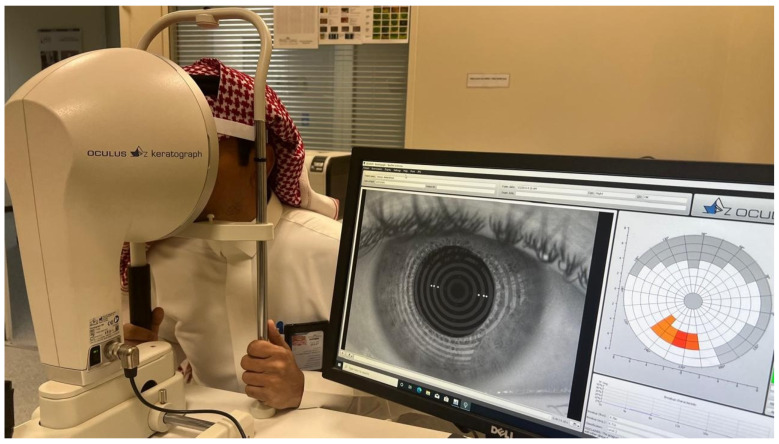
Photograph from a keratography device used to evaluate the tear film.

Data collection used standardized methods and tools to ensure accurate and reliable findings. Participants were first asked to complete the Ocular Surface Disease Index (OSDI) questionnaire to evaluate DED symptoms and their impact on daily activities; the validated Arabic version of the Ocular Surface Disease Index (OSDI) questionnaire was used in this study [11]. Advanced diagnostic tools were employed to obtain objective measurements. The Pentacam device (Oculus; Optikgeräte GmbH, Wetzlar, Germany) was used to assess key corneal parameters, including Kmax readings and the thinnest corneal area, while the Keratograph 5M (Oculus Optikgeräte GmbH, Wetzlar, Germany) provided detailed information on non-invasive tear breakup time, tear meniscus height, Meibomian gland morphology, and lipid layer integrity, as shown in Figure 1. Both devices were calibrated daily according to manufacturer instructions. All patient assessments were conducted between 12:00 PM and 2:00 PM to maintain consistency in the measurement conditions.

Additional clinical data, including participants’ age, gender, and ocular health history, were collected through structured interviews and a review of medical records to control for potential confounding factors. Ethical considerations were central to the study design. All participants provided informed consent before their involvement, and their confidentiality and privacy were strictly maintained throughout the research process. Participants retained the right to withdraw from the study or request the deletion of their data at any time without providing a reason.

The collected data were statistically analyzed using the Shapiro–Wilk test to assess data normality, and all the datasets failed the normality test according to the Shapiro–Wilk assessment; therefore, non-parametric tests (Wilcoxon rank-sum) were used for group comparisons. A *p*-value of less than 0.05 was considered statistically significant. By improving our understanding of the epidemiology of DED and its association with KC, with our findings, we aim to advance KC research and contribute to alleviating the discomfort experienced by affected patients.

## 3. Results

The participants’ ages ranged from 18 to 35 years, with a mean age of 23.8 ± 9.5 years. The key corneal topography parameters and dry eye test results are summarized in Table 1.

**Figure 2:**
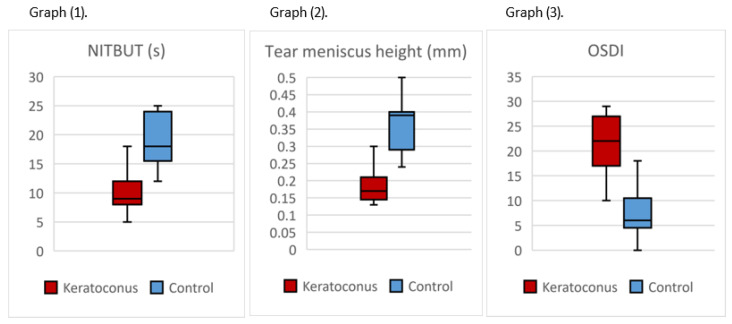
Graph 1 shows the comparison of keratoconus and control groups for NITBUT, Graph 2 for tear meniscus height (TMH), and Graph 3 for OSDI score.

Dry eye assessment showed that the mean non-invasive tear breakup time (NITBUT) was significantly shorter in the KC group (9.88 ± 3.25 s) compared to the control group (18.94 ± 4.26 s, *p* < 0.001; 95% CI: 6.7–11.3). Tear meniscus height was also significantly reduced in the KC group (0.185 ± 0.053 mm) versus the control group (0.358 ± 0.076 mm, *p* < 0.001; 95% CI: 0.13–0.23). The OSDI questionnaire scores were notably higher in the KC group (21.41 ± 5.93) than in the control group (7.52 ± 5.05, *p* < 0.001; 95% CI: 10.1–17.7) as shown in Figure 2.

Meibography findings revealed a higher grade of Meibomian gland dropout in the KC group (2.11 ± 0.60) compared to the control group (0.70 ± 0.34, *p* < 0.001) as shown in Table 2. However, no statistically significant difference was observed between the two groups in lipid layer evaluation, with the mean lipid layer grade being 1.65 ± 0.93 in the KC group and 1.12 ± 0.60 in the control group (*p* = 0.070).

### Relationship Between Parameters

**Figure 3:**
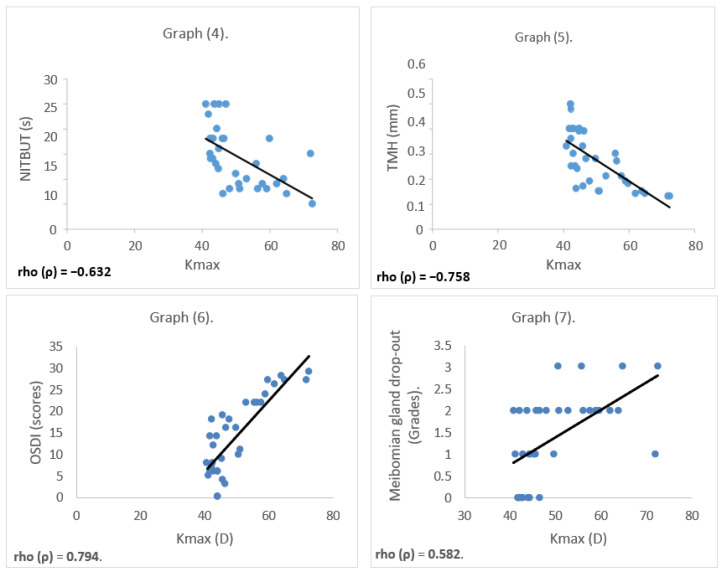
Graph 4 shows the correlations between Kmax and non-invasive tear breakup time (NITBUT) (*p* = −0.632). Graph 5 shows the correlation between Kmax and tear meniscus height (TMH) (*p* = −0.758). Graph 6 shows the correlation between Kmax and OSDI score (*p* = 0.794). Graph 7 shows the correlation between Kmax and meibomian gland drop-out grade (*p* = 0.582).

Correlation analyses were conducted only for the parameters that showed statistically significant differences between the groups. Spearman’s rho test revealed significant correlations between clinical parameters. Kmax showed a significant negative correlation with NITBUT and TMH (*p* < 0.001), and a significant positive correlation with OSDI and Meibomian gland drop-out (*p* < 0.001), as shown in Figure 3.

## 4. Discussion

KC has traditionally been regarded as a non-inflammatory ectatic corneal disorder due to the absence of classical signs of inflammation. However, recent evidence has challenged this assumption. Elevated levels of inflammatory mediators—including interleukins, cytokines, and proteolytic enzymes—have been identified in the tears and on the ocular surface of patients with KC [12,13,14,15,16,17,18], suggesting that subclinical inflammation plays a role in the disease’s pathophysiology. Elevated tear levels of IL-6 and MMP-9 have been reported in both KC and dry eye, suggesting shared inflammatory pathways influencing corneal remodeling and tear instability [19,20,21]. Similarly, DED is now recognized as a multifactorial condition in which inflammation plays a central role [15,16]. Given the overlap in inflammatory mechanisms, the potential association between KC and DED has attracted growing interest. In the present investigation, the presence of tear film instability and ocular surface disruption in individuals with KC was examined in comparison to healthy controls.

Significant alterations were identified in several dry eye parameters among KC patients. Non-invasive tear breakup time (NITBUT) and tear meniscus height values were notably reduced, while Ocular Surface Disease Index (OSDI) scores and Meibomian gland dropout grades were elevated relative to control subjects. These findings point to a greater prevalence of dry eye-related signs and symptoms in the KC population. In contrast, no significant differences were observed in lipid layer thickness, suggesting that evaporative dry eye mechanisms may be less prominent or variable across individuals with KC. The relationship between KC and DED remains incompletely understood, with previous studies reporting inconsistent results. Some investigations have documented reduced TBUT and Schirmer-I values among KC patients, with varying levels of statistical significance [2,3,13,22]. Differences in sample size, disease severity, and diagnostic methodology may contribute to this variability. While certain studies reported insignificant changes in TBUT, others have demonstrated reduced tear production and corneal sensitivity, particularly in more advanced stages of the disease [2,3,22]. The implications of each key parameter (e.g., tear film stability, ocular surface staining, and symptom severity) have been discussed in relation to patient management and disease understanding. Conventional tests like fluorescein tear breakup time and the Schirmer test can disturb natural tear flow and affect accuracy [23,24]. In contrast, our results show that keratography provides a reliable, contact-free, and objective way to assess tear stability, production, and meibomian gland function [25,26]. Growing evidence supports the use of non-invasive imaging tools to improve both patient comfort and the consistency of clinical assessments [27,28,29].

In the current findings, corneal steepening (Kmax) showed significant correlations with key ocular surface parameters. Increased Kmax values were associated with reduced tear film stability (as indicated by lower NITBUT and tear meniscus height), higher symptom burden (reflected in elevated OSDI scores), and greater Meibomian gland dropout. These correlations support the hypothesis that greater disease severity in KC may be linked to more pronounced ocular surface dysfunction. Overall, these observations contribute to a growing body of literature suggesting that inflammatory activity and tear film instability may play a more prominent role in KC than previously assumed. Addressing dry eye symptoms in KC not only enhances visual comfort but may also improve patients’ daily functioning and overall quality of life. Comprehensive assessment of the ocular surface, including dry eye evaluation, appears warranted in the clinical management of KC.

## 5. Conclusions

In conclusion, the findings indicate an association between KC and ocular surface changes consistent with features of dry eye disease. Evidence of tear film instability, ocular surface symptoms, and Meibomian gland dysfunction in KC patients underscores the relevance of dry eye evaluation in this population. Keratoconus and dry eye disease frequently occur together, yet non-invasive diagnostic techniques are still not widely used in clinical practice. The present study demonstrates that keratography provides an effective, tear-preserving approach to assessing ocular surface health in these patients. These insights suggest that addressing ocular surface health may play a role in the broader clinical management of KC and highlight the importance of further research into the underlying mechanisms linking these conditions.

## Figures and Tables

**Table 1:** Clinical characteristics of the groups.

	Mean ± SD		*p*
	Keratoconus Group	Control Group	
Age (years)	25.9 ± 6.8	22.6 ± 8.4	0.890
Kmax (dioptry)	56.85 ± 8.41 D	43.59 ± 1.81 D	<0.0001
Thinnest Corneal thickness (µm)	419.52 ± 58.61 µm	535.47 ± 38.34 µm	<0.0001
NITBUT (s)	9.88 ± 3.25 s	18.94 ± 4.26 s	<0.0001
TMH (mm)	0.185 ± 0.053 mm	0.358 ± 0.076 mm	<0.0001
OSDI score	21.41 ± 5.93	7.52 ± 5.05	<0.0001

**Table 2:** Meibomian gland drop-out grades of the groups.

	Mean ± SD		*p*
	Keratoconus Group	Control Group	*p*
Grade 0 (n)	0	8	<0.001
Grade 1 (n)	2	6	<0.001
Grade 2 (n)	11	3	<0.001
Grade 3 (n)	4	0	<0.001

Meibomian glands were scored using the following grades: Grade 0: No loss of meibomian glands. Grade 1: Area loss was less than one-third of the total meibomian gland area. Grade 2: Area loss was between one-third and two-thirds. Grade 3: Area loss was more than two-thirds.

## Data Availability

Data are available on request from the corresponding author. The data are not publicly available due to privacy and ethical restrictions.
